# Effects of Dietary Supplementation with Conjugated Linoleic Acid on Redox Marker Profiles and Neuroaxonal Degeneration in Amyotrophic Lateral Sclerosis

**DOI:** 10.3390/antiox15091194

**Published:** 2026-09-18

**Authors:** Minoo Sharbafshaaer, Roberta Pepe, Elvira Cortini, Teresa Biscardi, Rosaria Notariale, Nicolò Mesini, Elisabetta Zucchi, Jessica Mandrioli, Alessandro Tessitore, Paolo Bergamo, Francesca Trojsi

**Affiliations:** 1 Neurology Unit, First Division of Neurology and Neuro-Physiopathology, AOU University of Campania “Luigi Vanvitelli”, 80138 Naples, Italy; minoo.sharbafshaaer@unicampania.it (M.S.); notarialer@gmail.com (R.N.); alessandro.tessitore@unicampania.it (A.T.); francesca.trojsi@unicampania.it (F.T.); 2 Department of Advanced Medical and Surgical Sciences (DAMSS), University of Campania “Luigi Vavitelli”, 80138 Naples, Italy; roberta.pepe@unicampania.it; 3 Institute of Biosciences and Bio-Resources, National Research Council (CNR-IBBR), 80100 Naples, Italy; elviracortini1@gmail.com (E.C.); teresa.biscardi.tb@gmail.com (T.B.); 4 Department of Biomedical, Metabolic and Neural Sciences, University of Modena and Reggio Emilia, 41121 Modena, Italy; nicolomesini@hotmail.it (N.M.); zucchi.elisabetta@aou.mo.it (E.Z.); jmandrio@unimore.it (J.M.); 5 Department of Neurosciences, Azienda Ospedaliero Universitaria di Modena, 41124 Modena, Italy

**Keywords:** amyotrophic lateral sclerosis, neurofilament light chain, conjugated linoleic acid, ALSFRS-R, neurodegeneration, systemic redox biomarkers, Nrf2-mediated redox homeostasis

## Abstract

The revised ALS Functional Rating Scale (ALSFRS-R) and the blood plasma accumulation of neurofilament light chain (NfL) are key markers for tracking amyotrophic lateral sclerosis (ALS) progression. Based on recent studies indicating that dietary supplementation with conjugated linoleic acid (CLA) may boost antioxidant enzyme activity in ALS patients, in this study, ALSFRS-R scores (and subscores), NfL and thiol-bound protein (P-SH) levels, and intracellular activity of antioxidant enzymes (G6PD and GSR) were measured in ALS patients after 6 months (T6) of treatment with riluzole (R) or riluzole + CLA (R + CLA); we also investigated the correlations between these markers. At baseline (T0), G6PD activity in the R group was positively correlated with ALSFRS-R score (*p* < 0.001), whereas plasma P-SH levels in the R + CLA group were inversely correlated with ALSFRS-R score (*p* = 0.033). No significant baseline association was found between NfL levels and total ALSFRS-R score in the R group. At six months (T6), GSR and G6PD activities in the R + CLA group were significantly higher than in the R group (*p* < 0.05). Furthermore, a significant inverse correlation between plasma NfL levels and ALSFRS-R total score emerged at T6 exclusively in the R group (*p* = 0.002). Subscore analysis at T6 showed that plasma NfL levels negatively correlated with bulbar function in both groups (R: *p* = 0.010; R + CLA: *p* = 0.020) and with fine motor function in the R group (*p* = 0.031); whereas no significant correlation was observed for the respiratory subscore in either treatment group. These findings are consistent with an enhanced antioxidant activity associated with CLA supplementation. Overall, these preliminary exploratory results suggest that systemic redox biomarkers and plasma NfL may provide complementary, non-redundant information on distinct aspects of ALS pathobiology, though their precise temporal and mechanistic interactions require confirmation by dedicated longitudinal studies. The need for the combined use of neurodegeneration and oxidative-stress biomarkers for monitoring the progression of ALS is highlighted.

## 1. Introduction

Amyotrophic lateral sclerosis (ALS) is a progressive and fatal neurodegenerative disorder characterized by selective degeneration of upper and lower motor neurons, ultimately leading to progressive functional decline and reduced survival. Despite significant advances in genetics, biomarker discovery, and disease modeling, ALS remains a neurobiologically heterogeneous condition in which multiple interconnected pathogenic processes converge, including oxidative stress, mitochondrial dysfunction, RNA metabolism dysregulation, impaired proteostasis, and neuroimmune activation [1,2,3,4]. Growing evidence suggests that these mechanisms do not act independently but instead form a dynamic, interdependent network that drives disease initiation and progression. Within this integrated pathogenic landscape, redox dysregulation has emerged as a central and systemically relevant component of ALS pathobiology. An imbalance between the production of reactive oxygen species (ROS) and the capacity of endogenous antioxidant defense systems may result in a persistent pro-oxidative state, leading to cumulative damage to lipids, proteins, and nucleic acids [5,6,7]. Motor neurons are particularly vulnerable to oxidative stress due to their high metabolic demand, extensive axonal architecture, and limited regeneration capacity, making them especially sensitive to disturbances in redox homeostasis [8]. Importantly, recent studies have highlighted that oxidative stress in ALS extends beyond the central nervous system and involves peripheral biological compartments, including blood-derived cells and plasma, where reproducible alterations in redox-related pathways have been observed [9,10,11]. In this context, nuclear factor erythroid 2-related factor 2 (Nrf2) has been recognized as the main transcriptional activator of genes responsible for maintaining cellular redox homeostasis, and its lower efficacy in ALS patients/animal models was first demonstrated in 2008 [12,13,14]. In particular, the growing body of evidence indicating an association between ALS pathogenesis and impaired Nrf2-activated defenses [15,16] has highlighted this transcription factor as a potential therapeutic target for ALS [17]. The different antioxidant enzymes modulated by the Nrf2 pathway, such as glutathione reductase (GSR) and glucose-6-phosphate dehydrogenase (G6PD), in red blood cells (RBCs) play a pivotal role in the homeostasis of reduced glutathione (GSH), which is the most abundant intracellular antioxidant [18,19]. Similarly, circulating thiol-bound proteins (P-SHs) represent a critical component of the systemic redox buffering capacity and is a reliable redox status marker [20,21,22]; its levels along with the antioxidant enzyme activity in RBCs have been recognized as integrative markers of unbalanced redox homeostasis in cerebrospinal fluid/red blood cells (RBCs) of ALS patients [23,24,25]. However, the relationship between systemic redox alterations and clinical disease progression, particularly across distinct disease stages and domain-specific functional domains, remains insufficiently understood.

Plasma and cerebrospinal fluid levels of neurofilament light chain (NfL), a structural protein of the neuronal cytoskeleton that is released upon axonal injury, have emerged some of the most robust and reproducible fluid biomarkers of neuroaxonal damage [26,27]. Elevated NfL levels in plasma and cerebrospinal fluid have been consistently associated with disease severity, rate of progression, and survival outcomes in ALS, and are being increasingly used as pharmaco-dynamic and prognostic markers in clinical trials [28,29,30]. However, NfL primarily reflects downstream neurodegenerative processes and does not directly provide information on upstream pathogenic mechanisms such as redox imbalance or metabolic dysfunction. This raises a crucial question as to whether neuroaxonal degeneration and systemic redox biochemical alterations evolve in a coordinated manner or represent independent phenomena during disease progression. Against this background, growing attention has been directed toward the potential role of metabolic and nutritional interventions in modulating disease-related pathways in ALS. Conjugated linoleic acid—a food supplement consisting of an equimolar mixture of the cis-9, trans-11 and trans-10, cis-12 isomers of linoleic acid (CLA)—has been reported to be associated with antioxidant, anti-inflammatory, and metabolic regulatory effects across a range of experimental and clinical conditions [31,32]. Previous evidence in animal models of neurodegenerative disease indicated that dietary CLA may support redox homeostasis via the modulation of mitochondrial function and oxidative signaling pathways. Recent evidence suggests that CLA may influence redox homeostasis through modulation of lipid metabolism, mitochondrial function, and oxidative signaling pathways [33,34]. Notably, despite these properties, the potential role of CLA in modulating the interplay between neuroaxonal injury, clinical progression, and systemic redox status in ALS has not been investigated before. From a clinical perspective, the identification of reliable biomarkers capable of detecting disease activity and monitoring disease progression remains a major unmet need in ALS research. Based on these considerations, and on our previous proof-of-principle findings suggesting that conjugated linoleic acid (CLA) supplementation enhances Nrf2-mediated systemic antioxidant defenses in ALS patients [35], the present study was designed to explore the changes in plasma NfL levels, clinical status (assessed by the ALS Functional Rating Scale—Revised [ALSFRS-R]), and systemic redox biomarker measures over time and their associations in patients with ALS who were (or were not) treated with CLA supplementation. Specifically, we evaluated a dedicated subset of patients with paired plasma neurofilament light chain (NfL) and redox profiles to explore cross-sectional and timepoint-specific associations between plasma NfL levels, systemic redox biomarkers, and clinical functional status at T0 and T6 to analyze systemic oxidative stress, axonal degeneration, and domain-specific functional decline (ALSFRS-R subscales). By integrating clinical and biochemical parameters, this study aimed to clarify whether neuroaxonal damage and peripheral redox alterations are mechanistically linked or represent distinct biological axes of disease progression to ultimately inform future biomarker frameworks and therapeutic strategies targeting redox pathways in ALS.

## 2. Materials and Methods

### 2.1. Study Design and Participants

This observational study was conducted on a cohort of 49 patients diagnosed with ALS, who consecutively underwent clinical evaluation and screening at the University of Campania “Luigi Vanvitelli” (Naples, Italy) in September 2024. These ALS patients met the following criteria: classical, bulbar, upper motor neuron, or lower motor neuron phenotype [35]; symptoms related to the onset of the disease not earlier than 36 months after enrollment; and age at disease onset of 40 years or older. Patients were categorized into two treatment groups: one group received riluzole alone (100 mg/day) starting at diagnosis (R; n = 24), and the other group received riluzole combined with CLA supplementation (R + CLA; n = 25) starting from 3 months. Patients in the R + CLA group received 3 g/day of CLA orally, as in previous human studies. Disability status was assessed by evaluating the ALSFRS-R score (range: 0–48, with lower total scores reflecting greater disability) [36]. Clinical and demographic characteristics, including age, sex, disease duration, and total ALSFRS-R and subscale scores, together with blood samples, were collected at baseline (T0) and after 6 months of treatment (T6). Plasma NfL levels and selected systemic redox status measures were assessed to characterize their longitudinal changes and to examine their associations with functional disability measures. Treatment adherence was monitored during scheduled clinical evaluations through patient reporting, and no treatment discontinuation related to CLA supplementation occurred throughout the study period. At each timepoint, clinical data were recorded, and peripheral blood samples were collected for biomarker analyses, allowing for timepoint-specific and cross-sectional evaluation of candidate biomarkers. The research was conducted according to the principles expressed in the Declaration of Helsinki. Ethics approval was obtained from the Ethics Committee of the University of Campania “Luigi Vanvitelli” (Prot. No. 18192/2025), and written informed consent was obtained from all participants.

Regarding systemic redox marker values (G6PD, GSR, P-SHs), of the 49 ALS patients considered in this study (R: n = 24; R + CLA: n = 25), part of the cohort (R: n = 16; R + CLA: n = 20) overlapped with the cohort used in our previous study (35); the remaining 13 patients (R: n = 8; R + CLA: n = 5) represent newly recruited individuals analyzed under identical conditions. In contrast, plasma NfL values, domain-specific ALSFRS-R subscores and cross-sectional correlation analyses between neuroaxonal damage and redox profiles are herein presented for the first time.

### 2.2. Blood Collection and Processing

Biological sample preparation: Following collection, blood samples were centrifuged (1200 *g* for 10 min at 4 °C), and the plasma was separated, aliquoted, and stored at −80 °C until analysis. Immediately before analysis, plasma aliquots were diluted (1:100), and protein concentration was determined using the Bio-Rad Protein Assay Kit(Hercules, CA, USA). RBCs were prepared according to a previously published protocol [37] and stored in aliquots at −80 °C until use. Before analysis, RBC aliquots (approximately 30 mg) were resuspended in 250 µL of 20 mM Tris-HCl and 120 mM NaCl buffer (pH 7.0; TBS) supplemented with 0.1% Triton X-100 and 1.0 mM phenylmethylsulfonyl fluoride (PMSF) (TTBS), followed by incubation for 15 min at 4 °C. Cellular stroma was removed by centrifugation (300 *g* for 5 min at 4 °C), and the resulting supernatants were diluted in TBS (1:50). Protein concentration was subsequently determined using the Bio-Rad Protein Assay Kit.

### 2.3. Biomarker Assessment

Plasma neurofilament light chain levels were assessed as a marker of neuronal damage. Plasma samples were obtained by venipuncture, processed as previously reported [38], and stored in polypropylene tubes at −80 °C until analysis. All samples were centralized and finally processed for biomarker quantification in the Modena laboratory with a semi-automated immunoassay, the Ella™ microfluidic platform (BioTechne, Protein-Simple, San Jose, CA, USA), which has already been tested for measuring neurofilament levels in patients with ALS and other neurodegenerative diseases [39,40]. In parallel, systemic redox status was evaluated through the measurement of selected biomarkers reflecting key components of the enzymatic/non-enzymatic antioxidant defense systems (G6PD and GSR/circulating P-SHs). The latter are considered a robust systemic biomarker of redox status [19, 24] while G6PD and GSR activities in RBCs reflect the capacity to maintain cellular redox homeostasis, i.e., maintaining the levels of reduced glutathione, which is the main intracellular antioxidant. All biochemical analyses were performed using standardized spectrophotometric methods [35] and the results were normalized to protein content where appropriate.

### 2.4. Statistical Analysis

Statistical analyses were performed using SPSS software (version 27.0; IBM Corp., Armonk, NY, USA). Continuous variables are reported as the mean ± standard deviation (SD), while categorical variables are presented as frequencies and percentages. Comparisons between the two treatment groups were conducted using independent-samples Student’s *t*-tests for continuous variables and Pearson’s chi-square (*χ*^2^) tests for categorical variables. Correlation analyses were performed to investigate the relationships between plasma NfL levels, clinical status assessed by ALSFRS-R (total score and subscores), and redox-related biomarkers (G6PD, GSR, and P-SHs), using Spearman’s rank correlation coefficient (*ρ*). Values at T0 and T6 are reported descriptively. Correlation analyses were evaluated cross-sectionally at each specific timepoint rather than as formal within-subject changes or longitudinal time × treatment-group interactions. All analyses were stratified by treatment group and timepoint (T0 and T6) to explore potential time-dependent and treatment-specific patterns. All statistical tests were two-tailed, and a *p*-value < 0.05 was considered statistically significant. Given the exploratory, cross-sectional and hypothesis-generating nature of the study, correlation analyses across multiple biomarkers and clinical subscales were evaluated without primary adjustment for multiple comparisons. Consequently, observed associations with nominal significance (*p* < 0.05) are strictly interpreted as preliminary, nominal associations rather than definitive mechanistic links, and caution is exercised in drawing biological conclusions from isolated unadjusted *p*-values.

## 3. Results

### 3.1. Patient Characteristics and Clinical Features

A total of 49 patients with ALS were included in the study, comprising 24 patients treated with riluzole alone (R) and 25 patients treated with riluzole combined with conjugated linoleic acid supplementation (R + CLA). The overall cohort had a mean age of 60.12 ± 9.10 years and was predominantly male (31/49, 63.3%). The mean disease duration was 32.92 ± 16.25 months, and bulbar onset was present in 11 patients (22.4%). The baseline demographic, clinical, and plasma NfL characteristics are summarized in Table 1. No significant between-group differences were observed for age (59.29 ± 9.26 vs. 60.92 ± 9.06 years, *p* = 0.537), sex distribution (50.0% vs. 76.0% male, *p* = 0.112), disease duration (31.67 ± 20.19 vs. 34.12 ± 11.58 months, *p* = 0.607), bulbar onset frequency (25.0% vs. 20.0%, *p* = 0.673), baseline ALSFRS-R score (39.17 ± 6.01 vs. 38.04 ± 5.30, *p* = 0.491), ALSFRS-R score at 6 months (34.58 ± 7.63 vs. 33.32 ± 6.70, *p* = 0.542), baseline plasma NfL levels (88.20 ± 49.29 vs. 91.59 ± 74.57 pg/mL, *p* = 0.851), or plasma NfL levels at 6 months (97.25 ± 62.08 vs. 94.06 ± 84.82 pg/mL, *p* = 0.881). ALSFRS-R scores and plasma NfL levels are reported descriptively at T0 and T6 for both treatment groups (Table 1).

### 3.2. Systemic Redox Biomarkers and Group Comparisons

Systemic redox parameters were evaluated cross-sectionally at T0 and T6. At baseline (T0), no significant differences were detected between the R and R + CLA groups in RBC G6PD activity, GSR activity, or plasma P-SH levels (all *p* > 0.05). At the 6-month evaluation (T6), patients in the R + CLA group exhibited significantly higher G6PD activity (mean ± SD: 14.8 ± 3.2 vs. 11.2 ± 2.8 U/g Hb, *p* = 0.018) and GSR activity (mean ± SD: 8.5 ± 1.9 vs. 6.8 ± 1.6 U/g Hb, *p* = 0.032) compared to the R group, alongside preserved plasma P-SH levels (*p* = 0.038). These redox findings build upon and extend our earlier subset observations (35) with the current larger cohort.

### 3.3. Significant Associations Between Blood Biomarkers and Total ALSFRS-R Score

**Figure 1:**
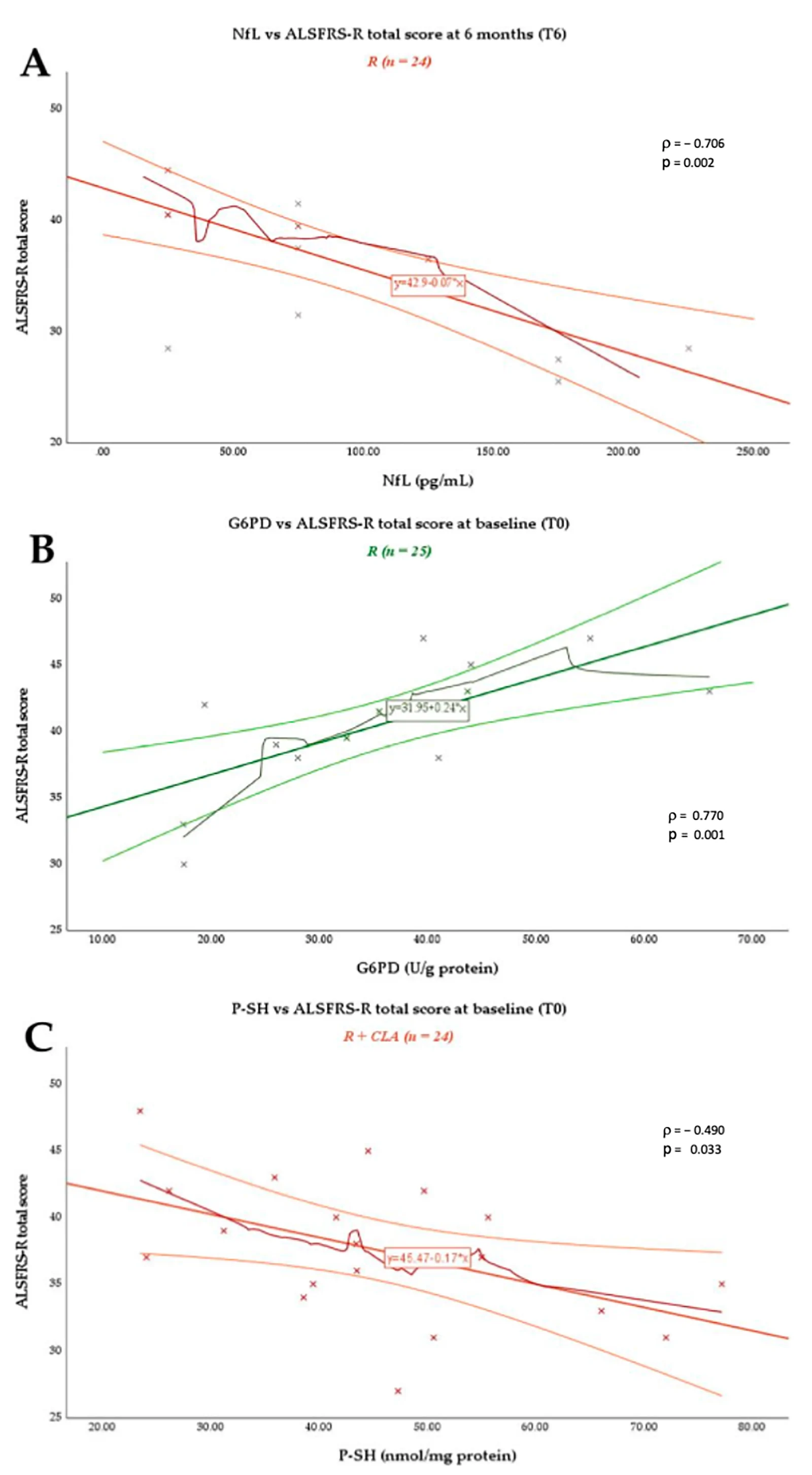
Cross-sectional associations between (**A**) plasma NfL and total ALSFRS-R score in the R group at T6; (**B**) G6PD activity and total ALSFRS-R score in the R group at T0; and (**C**) P-SH levels and total ALSFRS-R score in the R + CLA group at T0. Points represent individual data. Lines represent linear regression fits with 95% confidence intervals. Spearman’s correlation coefficients and two-sided unadjusted *p*-values are reported. *, ** *p* < 0.05, <0.01.

Cross-sectional correlation analyses revealed distinct biomarker-specific relationships with functional status across the treatment groups at each specific study timepoint (T0 and T6) (Figure 1). In the R group, plasma NfL levels were not significantly associated with ALSFRS-R total score at baseline (T0 *ρ* = −0.269, *p* = 0.313). At T6, a strong inverse relationship was observed (*ρ* = −0.706, *p* = 0.002), where higher circulating NfL concentrations corresponded to lower functional performance (Figure 1A). No significant correlation was detected in the R + CLA group at either timepoint. A different pattern was observed for G6PD activity. At baseline (T0), G6PD showed a robust positive association with ALSFRS-R total score in the R group (*ρ* = 0.770, *p* < 0.001), representing the strongest biomarker–clinical correlation identified in the cohort (Figure 1B). At T6, this association was not present (*ρ* = −0.021, *p* = 0.940). In the R + CLA group, plasma P-SH levels showed a moderate inverse association with ALSFRS-R total score at baseline (T0 *ρ* = −0.490, *p* = 0.033) (Figure 1C). At T6, no significant correlation was observed (*ρ* = −0.153, *p* = 0.531). No statistically significant associations were observed between GSR activity and ALSFRS-R total score in any treatment groups at T0 or T6.

### 3.4. Association Between Plasma NfL Levels and Functional Status

**Figure 2:**
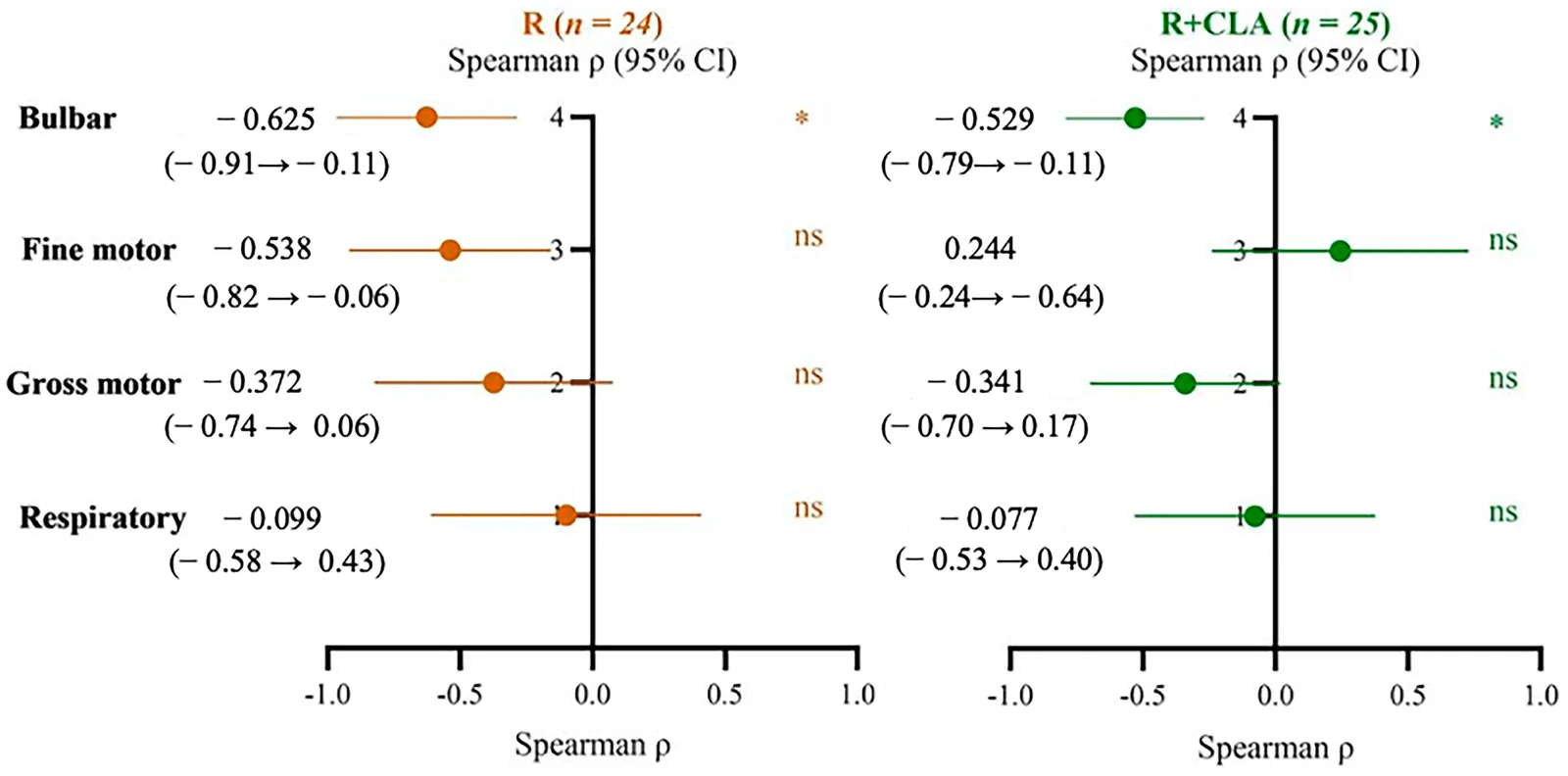
Correlations between plasma NfL levels and ALSFRS-R subscores at T6. Forest plots show Spearman correlation coefficients (*ρ*) and 95% confidence intervals for the bulbar, fine motor, gross motor, and respiratory ALSFRS-R subscores in patients in the R and R + CLA groups. Significant correlations are indicated by * *p* < 0.05; ns: not significant.

To further characterize the relationship between neuroaxonal injury and specific functional domains, associations between plasma NfL levels and ALSFRS-R subscores were evaluated at the 6-month follow-up (Figure 2). In the R group, plasma NfL levels showed a nominal inverse association with bulbar function (*ρ* = −0.625, 95% CI: −0.91 to −0.11, *p* = 0.010) and a preliminary nominal association with fine motor function (*ρ* = −0.538, 95% CI: −0.82 to −0.06, *p* = 0.031). No significant associations were observed for gross motor (*ρ* = −0.372, 95% CI: −0.74 to 0.16, *p* > 0.05) or respiratory subscores (*ρ* = −0.099, 95% CI: −0.58 to 0.43, *p* > 0.05). In the R + CLA group, a nominal inverse association was identified between plasma NfL levels and bulbar function (*ρ* = −0.529, 95% CI: −0.79 to −0.11, *p* = 0.020). In contrast, no significant relationships were observed for fine motor (*ρ* = 0.244, 95% CI: −0.24 to 0.64), gross motor (*ρ* = −0.341, 95% CI: −0.70 to 0.17), or respiratory function (*ρ* = −0.077, 95%CI: −0.53 to 0.40) (all *p* > 0.05). Overall, bulbar function was the only ALSFRS-R subdomain that showed consistent nominal association with plasma NfL levels in both treatment groups, whereas associations with the other functional domains were either absent or restricted to the R group.

### 3.5. Association Between Plasma NfL Levels and Functional Status

**Figure 3:**
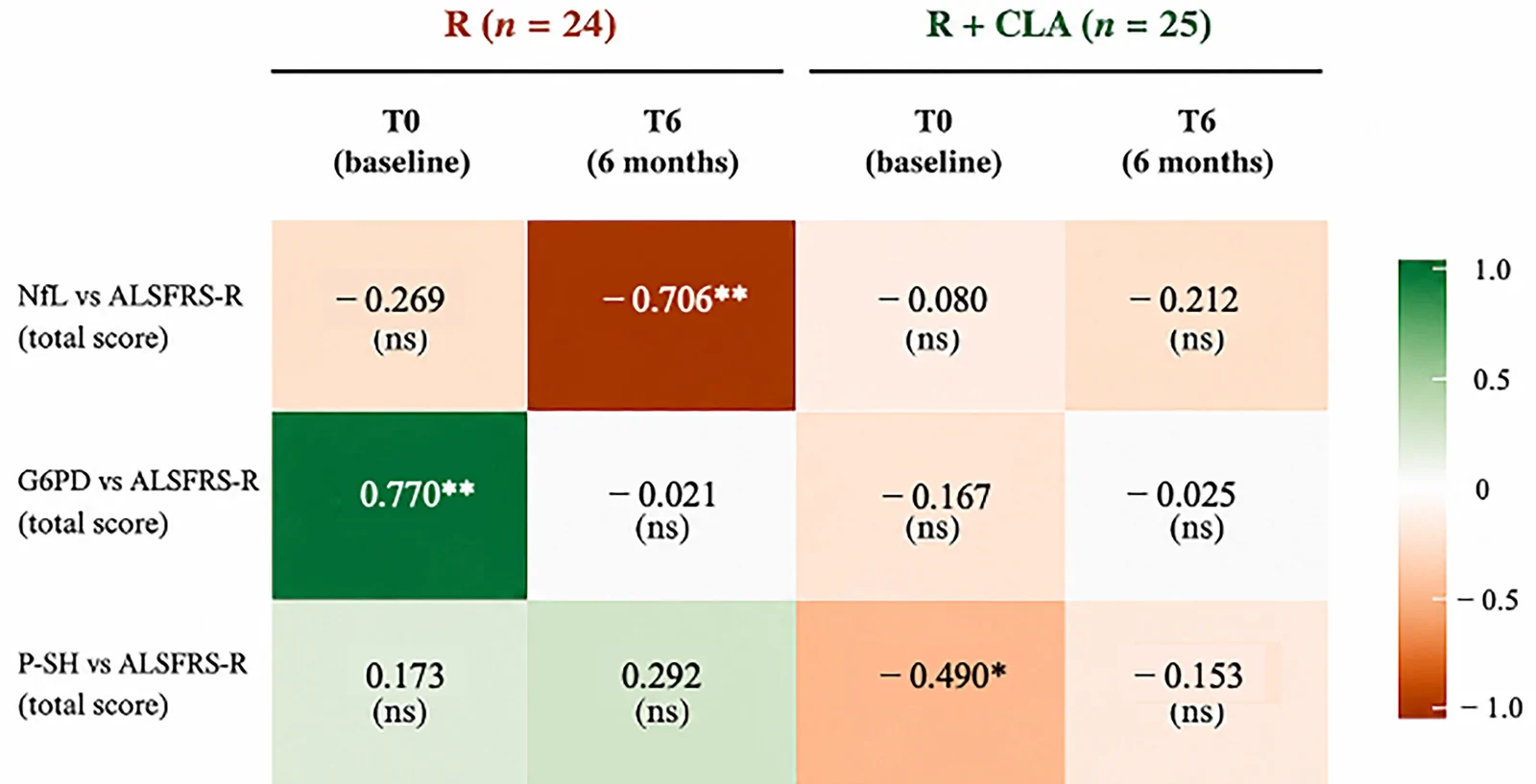
Integrated correlation heatmap summarizing associations between plasma NfL, redox biomarkers (G6PD and P-SHs), and total ALSFRS-R score across treatment groups and study timepoints. Values represent Spearman correlation coefficients (*ρ*). Green indicates positive correlations, whereas orange/red indicates negative correlations. Statistical significance is indicated as * *p* < 0.05 and ** *p* < 0.01; ns: not significant. T0: baseline; T6: 6-month follow-up.

To integrate the relationships between neuroaxonal injury, systemic redox status, and functional disability, a correlation heatmap was generated summarizing the associations between plasma NfL, selected redox biomarkers (G6PD and P-SHs), and total ALSFRS-R score across treatment groups and study timepoints (Figure 3). Distinct biomarker-specific patterns emerged from this integrated analysis. In the R group, G6PD activity exhibited a strong positive correlation with total ALSFRS-R score at baseline (*ρ* = 0.770, *p* < 0.001), whereas plasma NfL levels showed a strong inverse correlation with total ALSFRS-R score at the 6-month follow-up (*ρ* = −0.706, *p* = 0.002). In contrast, in the R + CLA group, P-SH levels were inversely associated with total ALSFRS-R score at baseline (*ρ* = −0.490, *p* = 0.033), while no significant relationship was observed between NfL and total ALSFRS-R score at either assessment. Overall, the heatmap highlights that the nominal biomarker–clinical associations were not uniformly distributed across treatment groups or follow-up assessments. Rather, NfL and redox-related biomarkers exhibited distinct temporal association patterns with functional status, with significant correlations emerging at different timepoints and in different treatment groups.

## 4. Discussion

Although the etiological role of redox state deterioration in neurodegenerative diseases is widely recognized, the relationship between the biomarkers of these two pathophysiological mechanisms has scarcely been investigated. In particular, given recent data on the modulatory capacity of CLA on systemic markers of redox status in ALS patients, this study aimed to evaluate their correlation with the level of a well-established marker of neuroaxonal damage: NfL. Changes after 6 months were explored in ALS patients treated with R alone or R + CLA, integrating measurements of biomarkers of antioxidant defenses (GSR, G6PD, and P-SHs), neuroaxonal degeneration (NfL), and functional status (ALSFRS-R), to assess whether these biomarkers reflect interconnected aspects of disease biology or distinct dimensions of ALS pathology. Several key findings emerged. CLA supplementation was associated with significant increases in the activities of Nrf2-activated antioxidant enzymes (G6PD and GSR), consistent with a systemic redox response. In addition, NfL and redox biomarkers showed different timepoint-specific patterns of association with clinical disability. Taken together, these findings suggest that neuroaxonal damage and peripheral redox alterations may reflect complementary or partially distinct biological processes in ALS, although the present analyses do not allow their temporal relationship or independence to be established. These correlations should be strictly interpreted as cross-sectional, timepoint-specific relationships rather than evidence of longitudinal change, differential treatment effects, or distinct biomarker trajectories. A major observation of the present study was the negligible variability in plasma NfL concentrations over the 6-month follow-up period despite a measurable decline in ALSFRS-R scores. NfL is currently considered one of the most robust fluid biomarkers in ALS and has consistently been associated with disease severity, progression rate, and survival outcomes [22,23,24,25,26,27,28]. However, increasing evidence suggests that NfL concentrations often reach a relatively stable plateau after symptom onset, reflecting ongoing neuroaxonal degeneration rather than continuous linear increases during disease progression [22,28,41,42]. Our findings are therefore consistent with previous longitudinal investigations demonstrating limited intra-individual variation in circulating NfL levels over time despite clinical deterioration [22,28,41]. Nevertheless, since within-subject changes were not formally assessed in the present analysis, these findings should not be considered as evidence of individual NfL stability. Rather, the observed pattern is compatible with the interpretation that NfL primarily captures the biological burden of axonal injury rather than short-term fluctuations in functional performance. Although NfL concentrations remained stable at the group level, higher NfL levels were strongly associated with lower ALSFRS-R scores at follow-up in the R group. Furthermore, NfL demonstrated consistent inverse associations with bulbar function in both treatment groups and with fine motor function in the R group. These observations are consistent with previous studies showing that elevated NfL levels reflect greater neuroaxonal damage and more severe clinical impairment [26,27,28,29]. In contrast, no significant association was observed between NfL levels and overall ALSFRS-R scores in the R + CLA group at 6 months. This finding should be interpreted cautiously. Given the observational nature and the 6-month follow-up duration, CLA supplementation was associated with an enhancement of peripheral redox defenses, while no significant differences between groups were detected in plasma NfL concentrations or in the trajectory of clinical functional decline. This lack of effect on NfL levels is consistent with previous evidence showing that circulating NfL levels often reach a stable plateau after disease onset in ALS, making short-term changes difficult to detect over a 6-month timeframe. Rather than providing conclusive evidence of direct structural neuroprotection, the upregulation of G6PD and GSR activities reflects an early, adaptive systemic antioxidant response. A 6-month intervention period may be sufficient to detect systemic metabolic/redox adaptations but may not be long enough to evaluate potential downstream effects on neuroaxonal structural integrity or clinical progression. Future studies with longer follow-up are required to explore whether sustained modulation of systemic redox homeostasis can eventually influence neuroaxonal degeneration over extended periods. Along the same lines, the apparent reversal in the direction of the correlation between NfL levels and the ALSFRS-R fine motor subscore at follow-up in the R + CLA group may reflect a treatment-related modification of this relationship rather than a direct effect on neuroaxonal injury itself. Because these findings represent nominal, exploratory associations evaluated without formal adjustment for multiple comparisons, these biological interpretations remain speculative. Consequently, these preliminary nominal associations require validation in independent, larger longitudinal cohorts applying formal multiple-testing corrections (e.g., False Discovery Rate, FDR) and extended follow-up. Notably, the persistent inverse association between NfL and bulbar function in both treatment groups supports NfL as a marker of clinically meaningful neuroaxonal damage. The particularly strong relationship with bulbar dysfunction is biologically plausible given the recognized prognostic significance of bulbar involvement and its association with accelerated disease progression in ALS. In contrast to NfL levels, redox-related indicators demonstrated a distinct biological profile. The most prominent effect of CLA supplementation was the significant increase in G6PD and GSR activities after six months. These enzymes are central components of cellular antioxidant defense systems. G6PD represents the principal source of NADPH generation through the pentose phosphate pathway, whereas GSR maintains glutathione in its reduced and biologically active state, thereby supporting detoxification of reactive oxygen species and preservation of intracellular redox homeostasis [11,18]. The observed increase in G6PD and GSR activities therefore suggests the activation of adaptive antioxidant mechanisms in response to CLA supplementation, involving Nrf2-regulated antioxidant pathways [17,43,44]. This interpretation is consistent with previous experimental and clinical evidence revealing that CLA can modulate redox-sensitive signaling networks through cellular mechanisms activated downstream of the Nrf2 pathway (mitochondrial regulation and redox homeostasis) [30,31,32,35]. Notably, the findings also align with the recent study by Biscardi and colleagues, which demonstrated that CLA supplementation improves Nrf2-related systemic antioxidant responses in ALS patients [35]. Together, these observations strengthen the hypothesis that CLA ameliorates peripheral antioxidant defense systems even in the absence of detectable effects on neuroaxonal injury markers in ALS patients. This apparent dissociation may provide valuable insight into ALS pathobiology. Oxidative stress is widely recognized as a central contributor to motor neuron degeneration and interacts with mitochondrial dysfunction, neuroinflammation, RNA dysregulation, and impaired proteostasis within a complex pathogenic network [1,2,3,4,5,6,7,17,20]. NfL is a well-established biomarker of neuroaxonal injury and reflects structural axonal damage that has already occurred rather than the molecular processes driving disease progression [29]. In contrast, G6PD, GSR, plasma P-SHs, and BDNF provide complementary information on antioxidant capacity, redox homeostasis, and neurotrophic support, which may influence neuronal resilience before measurable changes in axonal integrity become evident. Therefore, the enhancement of systemic antioxidant markers following CLA supplementation in the absence of changes in circulating NfL suggests that peripheral redox status and neuroaxonal injury reflect distinct pathophysiological dimensions. The lack of change in plasma NfL indicates that CLA does not exert a direct short-term neuroprotective effect on axonal breakdown. Given that NfL kinetics in ALS typically display stability over short intervals and that systemic metabolic shifts occur rapidly, the 6-month follow-up window reflects different response dynamics between systemic redox modulation and neuroaxonal damage, which represent distinct biological aspects rather than a proven temporal dissociation. These correlations should be strictly interpreted as cross-sectional, timepoint-specific relationships rather than evidence of longitudinal change, differential treatment effects, or distinct biomarker trajectories. Longer-term studies are needed to determine whether sustained modulation of redox homeostasis ultimately translates into reduced neuroaxonal injury. This observation reinforces the concept that molecular and neurodegenerative biomarkers capture distinct yet complementary layers of ALS pathobiology. The integrated correlation analyses further support this interpretation. Significant associations involving redox biomarkers emerged, predominantly at baseline, whereas associations involving NfL were more evident at follow-up. The strong positive relationship between G6PD activity and ALSFRS-R scores at baseline suggests that systemic antioxidant capacity may correlate with functional status at specific evaluation points. Conversely, the inverse relationship between NfL and ALSFRS-R at follow-up indicates that neuroaxonal injury remains a key correlate of clinical disability. Rather than reflecting temporally distinct trajectories, these timepoint-specific associations suggest that redox dysregulation and neuroaxonal degeneration capture partially independent biological dimensions of ALS. From a broader perspective, our findings have important implications for biomarker development in ALS. Current biomarker strategies increasingly acknowledge that no single marker can adequately capture the biological complexity of the disease [1,2,3,4]. While NfL provides a sensitive measure of neuroaxonal injury and prognostic information [22,26,27,28,29], it offers limited mechanistic insight into upstream disease processes. Conversely, redox biomarkers may reflect potentially modifiable pathogenic pathways but do not directly quantify neuronal loss. The present findings therefore support the development of multidimensional biomarker frameworks integrating markers of neurodegeneration with indicators of oxidative and metabolic dysfunction. Such approaches may improve patient stratification, facilitate monitoring of mechanism-specific therapeutic interventions, and provide a more comprehensive representation of disease biology []. Several methodological limitations should be considered when interpreting these findings. First, this was an observational study with only two timepoints (T0 and T6) and a relatively modest sample size, which limits our ability to establish causality or definitively infer temporally distinct trajectories. Moreover, the non-randomized treatment allocation may have introduced residual confounding, and within-subject changes and group-by-time interactions were not formally assessed. Second, given the exploratory and hypothesis-generating nature of the correlation analyses, no formal adjustment for multiple testing was applied. Therefore, isolated unadjusted *p*-values (particularly those in the nominal 0.02–0.03 range) must be interpreted with caution, and biological speculation derived from them should be reduced until confirmed by sensitivity analyses using False Discovery Rate (FDR) corrections in larger cohorts. Correlations were also not formally compared across treatment groups or timepoints; therefore, differences in statistical significance should not be interpreted as evidence that the associations differed between groups or over time. Third, while CLA supplementation was associated with a systemic enhancement of Nrf2-mediated antioxidant enzyme activities (G6PD and GSR), this was not accompanied by significant between-group differences in plasma NfL concentrations or clinical functional status at T6. However, given the study design and statistical approach, this finding does not demonstrate the absence of a treatment effect or that peripheral redox enhancement cannot translate into neurostructural protection or clinical stabilization. Larger longitudinal cohorts incorporating multiple timepoints, repeated-measures or mixed-effects statistical models, and appropriate control for multiple comparisons are required to confirm whether long-term redox modulation is associated with changes in neuroaxonal damage in ALS. These findings suggest that redox dysregulation and neuroaxonal degeneration represent biologically interconnected but partially dissociable processes, capturing distinct pathophysiological dimensions of ALS. Future studies integrating longitudinal measurements of molecular, biochemical, and neurodegenerative biomarkers will be essential to clarify the mechanistic relationships between these pathways and to optimize biomarker-guided therapeutic strategies.

## 5. Conclusions

The present study demonstrates timepoint-specific association patterns between plasma NfL and functional status in ALS. In conclusion, while CLA supplementation was associated with an enhanced systemic antioxidant response (increased G6PD and GSR activities), it did not significantly impact plasma NfL levels or clinical progression over the 6-month study period. These associations should be strictly interpreted as cross-sectional, timepoint-specific relationships rather than evidence of longitudinal change, differential treatment effects, or distinct biomarker trajectories. Rather than reflecting distinct temporal trajectories, our findings suggest that systemic redox alterations and neuroaxonal degeneration capture partially dissociable biological aspects of ALS. Consequently, redox biomarkers should not be viewed as direct surrogates for neuroaxonal damage, but rather as complementary indicators of systemic metabolic homeostasis in ALS. An integrated evaluation of neurodegenerative and redox-related indicators may thus provide a useful, multidimensional framework for patient characterization. Ultimately, to overcome the limitations of isolated unadjusted *p*-values, larger longitudinal cohorts with extended follow-up, formal interaction analyses, and rigorous statistical adjustments for multiple testing (such as FDR-based corrections) are required to validate these nominal associations and further clarify the relationship between systemic oxidative stress and central neurodegeneration in ALS.

## Figures and Tables

**Table 1:** Baseline demographic, clinical, and NfL (pg/mL) characteristics of patients with amyotrophic lateral sclerosis, stratified by treatment group.

Variable	R (n = 24)	R + CLA (n = 25)	Total (n = 49)	Test	*p*-Value
Age, years	59.29 ± 9.26	60.92 ± 9.06	60.12 ± 9.10	*t*(47) = −0.62	0.537
Sex, male n (%)	12 (50.0)	19 (76.0)	31 (63.3)	*χ*^2^(1) = 2.53	0.112
Disease duration, months	31.67 ± 20.19	34.12 ± 11.58	32.92 ± 16.25	*t*(47) = −0.52	0.607
Onset site (bulbar), n (%)	6 (25.0)	5 (20.0)	11 (22.4)	*χ*^2^(1) = 0.18	0.673
ALSFRS-R at baseline	39.17 ± 6.01	38.04 ± 5.30	38.59 ± 5.63	*t*(47) = 0.69	0.491
ALSFRS-R at 6 months	34.58 ± 7.63	33.32 ± 6.70	33.94 ± 7.13	*t*(47) = 0.62	0.542
NfL at baseline (pg/mL)	88.20 ± 49.29	91.59 ± 74.57	89.93 ± 62.83	*t*(47) = −0.19	0.851
NfL at 6 months (pg/mL)	97.25 ± 62.08	94.06 ± 84.82	95.62 ± 73.80	*t*(47) = 0.15	0.881

Data are presented as mean ± standard deviation (SD) or number (%). Group comparisons were performed using Student’s *t*-test for continuous variables and Pearson’s chi-square (*χ*^2^) test for categorical variables. Abbreviations: ALSFRS-R, Amyotrophic Lateral Sclerosis Functional Rating Scale—Revised; NfL, neurofilament light chain; R, riluzole; CLA, conjugated linoleic acid; SD, standard deviation; df, degrees of freedom.

## Data Availability

All data and materials support the reported claims and comply with the standards for data transparency. Data will be made available on reasonable request.

## Abbreviations

The following abbreviations are used in this manuscript:

ALSFRS-R	Amyotrophic Lateral Sclerosis Functional Rating Scale—Revised
CLA	conjugated linoleic acid
GSR	glutathione reductase
G6PD	glucose-6-phosphate dehydrogenase
NfL	neurofilament light chain
P-SHs	thiol-bound proteins
R	riluzole
R + CLA	riluzole + CLA
